# Associations of TNFR1 with kidney function outcomes by age, gender, and baseline kidney function status: Data from the Heart and Soul Study

**DOI:** 10.1016/j.dib.2017.07.048

**Published:** 2017-07-26

**Authors:** Meyeon Park, Daniela Maristany, Debbie Huang, Michael G. Shlipak, Mary Whooley

**Affiliations:** a Division of Nephrology, University of California, San Francisco, San Francisco, CA, USA; b Department of Epidemiology and Biostatistics, University of California, San Francisco, San Francisco, CA, USA; c School of Medicine, University of California, San Francisco, San Francisco, CA, USA; d San Francisco VA Medical Center, San Francisco, CA, USA; e Department of Medicine, University of California, San Francisco, San Francisco, CA, USA

**Keywords:** Kidney disease, Biomarkers, Data

## Abstract

Tumor necrosis factor receptor type 1 (TNFR1) is associated with kidney disease and mortality risk in various populations [1], [2]. We evaluated associations of TNFR1 with mortality and mediators of this relationship in doi: 10.1016/j.atherosclerosis.2017.05.021. Whether or not these associations are influenced by age, gender, or baseline kidney function are not known. We evaluated associations of TNFR1 levels with measures of kidney function stratifying by these variables. Our outcomes included estimated glomerular filtration rate (eGFR) < 60 ml/min/1.73 m^2^, albumin to creatinine ratio (ACR) >30 mg/g, and rapid kidney function loss, defined as a change in eGFR of greater than 3% per year.

**Specifications Table**

**Table:** 

Subject area	*Medicine, Biology*
More specific subject area	*Vascular biology*
Type of data	*Tables*
How data was acquired	*Multivariable Poisson models*
Data format	*analyzed*
Experimental factors	*n/a*
Experimental features	*n/a*
Data source location	*San Francisco, CA*
Data accessibility	*Raw data will be provided to interested investigators with a signed data use agreement by contacting the authors directly.*

**Value of the data**

• Tumor necrosis factor receptors 1 (TNFR1) [1,2] and 2 (TNFR2) are cell membrane-bound receptors involved in apoptosis, inflammation, and immune host defense [3].
• The soluble TNF receptors are present in the sera of healthy individuals, and elevated levels occur in a variety of pathologic states including sepsis and autoimmune disorders [4].
• Associations between TNFR1 and kidney function outcomes are uniformly strong [2], [5], [6].

## 1 Data

Our data include three tables describing incident rate ratios for associations of quartiles of TNFR1 with three outcomes: eGFR < 60 ml/min/1.73 m^2^ (Table 1), ACR >30 mg/g (Table 2), and rapid kidney function loss by subgroups (age, gender, and baseline kidney function assessed by eGFR and ACR) (Table 3). Multivariable models were conducted adjusting for demographics (Model 1); comorbid conditions (Model 2), and either baseline ACR for the outcomes of eGFR < 60 and rapid kidney function loss or baseline eGFR for the outcome of ACR > 30 mg/g (Model 3). Table 4 provides the p-values for interaction for these associations by age, gender, and eGFR or ACR at baseline.

**Table 1:** Associations between baseline quartiles of TNFR1 and CKD assessed by eGFR < 60 ml/min/1.73 m^2^, by subgroups.

		**Q1**	**Q2**	**Q3**		**Q4**
			**OR (95% CI)**	**OR (95% CI)**		**OR (95% CI)**
**Men**						
	Model 1 (n=798)	Ref	2.31 (1.07, 5.01)	5.89 (2.89, 12.02)		13.41 (6.65, 27.07)
	Model 2 (n=730)	Ref	2.00 (0.93, 4.30)	4.65 (2.27, 9.54)		9.67 (4.73, 19.78)
	Model 3a (n=695)	Ref	2.86 (1.13, 7.22)	6.61 (2.71, 16.14)		13.04 (5.31, 32.02)
**Women**						
	Model 1 (n=182)	Ref	2.80 (0.76, 10.33)	4.94 (1.41, 17.33)		12.90 (4.11, 40.48)
	Model 2 (n=151)	Ref	1.72 (0.43, 6.84)	4.25 (1.14, 15.88)		8.99 (2.54, 31.79)
	Model 3a (n=138)	Ref	2.23 (0.45, 11.08)	5.22 (1.11, 24.61)		9.06 (1.98, 41.37)
**Age > 67 years**						
	Model 1 (n=482)	Ref	1.58 (0.77, 3.26)	3.19 (1.66, 6.15)		6.61 (3.55, 12.33)
	Model 2 (n=433)	Ref	1.38 (0.68, 2.76)	2.70 (1.44, 5.07)		5.01 (2.72, 9.23)
	Model 3a (n=408)	Ref	1.84 (0.82, 4.12)	3.47 (1.63, 7.40)		6.02 (2.84, 12.80)
**Age < =67 years**						
	Model 1 (n=482)	Ref	5.08 (1.10, 23.50)	16.18 (3.86, 67.80)		57.14 (14.29, 228.55)
	Model 2 (n=433)	Ref	3.55 (0.73, 17.33)	12.87 (2.93, 56.60)		39.33 (9.46, 163.45)
	Model 3a (n=408)	Ref	5.64 (0.66, 48.02)	23.26 (177.26)		64.88 (8.97, 469.28)
**ACR ≥ 30 mg/g**						
	Model 1 (n=150)	Ref	3.49 (0.53, 23.04)	5.95 (0.95, 37.17)		9.89 (1.60, 61.10)
	Model 2 (n=125)	Ref	2.05 (0.34, 12.53)	4.38 (0.77, 25.03)		7.23 (1.30, 40.16)
**ACR < 30 mg/g**						
	Model 1 (n=768)	Ref	2.89 (1.19, 7.01)	7.35 (3.20, 16.91)		16.50 (7.23, 37.65)
	Model 2 (n=708)	Ref	2.61 (1.10, 6.21)	6.30 (2.78, 14.27)		12.12 (5.27, 27.88)

Model 1: Adjusted for demographic factors (age, sex, race)

Model 2: Model 1 + comorbid conditions (smoking, BMI, history of hypertension, diabetes, MI, HF, ACEI/ARB use, beta-blocker use, HDL, triglycerides, hemoglobin A1c, LVEF, METs)

Model 3a: Model 2 + ACR

**Table 2:** Associations between baseline quartiles of TNFR1 and ACR >=30, by subgroups.

		**Q1**	**Q2**	**Q3**	**Q4**
			**OR (95% CI)**	**OR (95% CI)**	**OR (95% CI)**
**Men**					
	Model 1 (n=757)	Ref	2.00 (1.00, 4.00)	2.76 (1.39, 5.48)	6.25 (3.31, 11.79)
	Model 2 (n=698)	Ref	1.94 (0.89, 4.21)	2.00 (0.91, 4.41)	3.16 (1.43, 7.01)
	Model 3b (n=695)	Ref	1.88 (0.82, 4.31)	1.69 (0.72, 3.97)	2.00 (0.81, 4.94)
**Women**					
	Model 1 (n=165)	Ref	2.37 (0.76, 7.42)	2.37 (0.67, 8.42)	5.71 (2.10, 15.57)
	Model 2 (n=139)	Ref	1.47 (0.43, 5.07)	2.18 (0.54, 8.83)	3.30 (0.93, 11.69)
	Model 3b (n=138)	Ref	1.53 (0.45, 5.22)	2.53 (0.58, 10.99)	4.74 (1.35, 16.70)
**Age > 67 years**					
	Model 1 (n=456)	Ref	2.02 (0.76, 5.36)	1.97 (0.73, 5.34)	4.78 (1.96, 11.66)
	Model 2 (n=411)	Ref	1.84 (0.64, 5.33)	1.53 (0.51, 4.60)	2.68 (0.92, 7.85)
	Model 3b (n=408)	Ref	2.00 (0.59, 6.80)	1.45 (0.40, 5.24)	2.08 (0.57, 7.61)
**Age < =67 years**					
	Model 1 (n=466)	Ref	1.95 (0.91, 4.15)	3.25 (1.57, 6.72)	7.21 (3.77, 13.80)
	Model 2 (n=426)	Ref	1.45 (0.64, 3.27)	2.80 (1.27, 6.16)	3.81 (1.68, 8.65)
	Model 3b (n=425)	Ref	1.44 (0.63, 3.30)	2.72 (1.21, 6.09)	3.57 (1.36, 9.39)
**eGFR<60**					
	Model 1 (n=277)	Ref	2.04 (0.27, 15.67)	1.55 (0.21, 11.53)	2.69 (0.38, 19.21)
	Model 2 (n=244)	Ref	1.09 (0.13, 9.34)	1.03 (0.12, 8.81)	1.41 (0.18, 11.24)
**eGFR >=60**					
	Model 1 (n=641)	Ref	2.00 (1.04, 3.86)	2.66 (1.30, 5.43)	4.75 (2.30, 9.82)
	Model 2 (n=589)	Ref	1.60 (0.79, 3.25)	1.62 (0.75, 3.51)	1.91 (0.78, 4.69)

Model 1: Adjusted for demographic factors (age, sex, race)

Model 2: Model 1 + comorbid conditions (smoking, BMI, history of hypertension, diabetes, MI, HF, ACEI/ARB use, beta-blocker use, HDL, triglycerides, hemoglobin A1c, LVEF, METs)

Model 3b: Model 2 + eGFR

**Table 3:** Associations between baseline quartiles of TNFR1 and Rapid Loss in Kidney Function, by subgroups.

		**Q1**	**Q2**	**Q3**	**Q4**
			**OR (95% CI)**	**OR (95% CI)**	**OR (95% CI)**
**Men**					
	Model 1 (n=514)	Ref	1.26 (0.68, 2.35)	1.60 (0.84, 3.04)	3.79 (2.00, 7.16)
	Model 2 (n=477)	Ref	1.20 (0.64, 2.27)	1.41 (0.74, 2.69)	2.70 (1.39, 5.23)
	Model 3b (n=457)	Ref	1.10 (0.57, 2.11)	2.11 (0.64, 2.30)	2.25 (1.13, 4.46)
**Women**					
	Model 1 (n=113)	Ref	1.28 (0.39, 4.26)	0.96 (0.19, 4.71)	2.16 (0.64, 7.31)
	Model 2 (n=100)	Ref	1.18 (0.34, 4.13)	0.20 (0.03, 1.20)	0.79 (0.19, 3.26)
	Model 3b (n=93)	Ref	1.45 (0.49, 4.28)	0.16 (0.02, 1.31)	0.47 (0.08, 2.81)
**Age > 67 years**					
	Model 1 (n=291)	Ref	1.75 (0.70, 4.33)	1.73 (0.66, 4.55)	3.46 (1.33, 8.98)
	Model 2 (n=271)	Ref	1.84 (0.74, 4.56)	1.78 (0.68, 4.64)	2.94 (1.10, 7.83)
	Model 3b (n=257)	Ref	1.88 (0.69, 5.11)	1.74 (0.63, 4.81)	2.77 (0.97, 7.92)
**Age < =67 years**					
	Model 1 (n=336)	Ref	0.95 (0.45, 2.04)	1.30 (0.59, 2.85)	3.97 (1.98, 7.95)
	Model 2 (n=306)	Ref	0.84 (0.39, 1.78)	1.00 (0.46, 2.18)	2.51 (1.18, 5.33)
	Model 3b (n=293)	Ref	0.85 (0.39, 1.84)	0.94 (0.42, 2.12)	2.23 (1.03, 5.23)

Model 1: Adjusted for demographic factors (age, sex, race)

Model 2: Model 1 + comorbid conditions (smoking, BMI, history of hypertension, diabetes, MI, HF, ACEI/ARB use, beta-blocker use, HDL, triglycerides, hemoglobin A1c, LVEF, METs)

Model 3b: Model 2 + eGFR

**Table 4:** P-values for interaction.

interaction term	outcome eGFR<60	outcome ACR>30	outcome rapid loss
TNFR1*age	0.0004	0.59	0.3
TNFR1*gender	0.95	0.79	0.77
TNFR1*eGFRbaseline	n/a	0.96	n/a
TNFR1*ACRbaseline	0.71	n/a	0.53

## 2 Experimental design, materials and methods

The Heart and Soul Study was a prospective cohort study designed to investigate the effects of psychosocial factors on health outcomes in patients with stable ischemic heart disease (IHD) [7]. Participants were eligible if they had a history of myocardial infarction; angiographic evidence of ≥50% stenosis in ≥1 coronary vessels; evidence of exercise-induced ischemia by treadmill ECG or stress nuclear perfusion imaging; or a history of coronary revascularization. Participants were excluded if they were unable to walk one block, had an acute coronary syndrome within the previous six months, or were likely to move out of the area within three years. 1024 subjects were recruited from 12 outpatient clinics in the San Francisco Bay Area between 9/2000 and 12/2002. Participants were divided into quartiles of TNFR1 levels. TNFR1 levels were normally distributed in the population studied. We evaluated cross-sectional associations with baseline kidney function and with longitudinal rapid kidney function loss using Poisson regression. We compared rates of the outcomes of MI, HF, and mortality between quartile 4 versus quartile 1 using multivariable Poisson regression models. For all regression models, adjustment variables included demographic characteristics (age, sex, race); lifestyle characteristics (smoking, BMI); and comorbid conditions (history of hypertension, diabetes, MI, HF, ACEI/ARB use, beta-blocker use, HDL, triglycerides, hemoglobin A1c, LVEF, METs). Covariates were selected based on evaluation of known confounders of atherosclerosis and kidney disease. In analyses of rapid kidney function loss, we adjusted for the baseline value and additionally adjusted for ACR in the final model. We then performed subgroup analyses by gender, age, and baseline kidney function.

## Ethical approval

Institutional Review Boards at each site approved this study protocol. All participants provided written informed consent.
